# Inkjet Printing Based Mono-layered Photonic Crystal Patterning for Anti-counterfeiting Structural Colors

**DOI:** 10.1038/srep30885

**Published:** 2016-08-04

**Authors:** Hyunmoon Nam, Kyungjun Song, Dogyeong Ha, Taesung Kim

**Affiliations:** 1 Department of Mechanical Engineering, Ulsan National Institute of Science and Technology (UNIST), 50 UNIST-gil, Ulsan 44919, Republic of Korea; 2 Korea Institute of Machinery and Materials, 156, Gajeongbuk-Ro, Yuseong-Gu, Daejeon 34103, Republic of Korea

## Abstract

Photonic crystal structures can be created to manipulate electromagnetic waves so that many studies have focused on designing photonic band-gaps for various applications including sensors, LEDs, lasers, and optical fibers. Here, we show that mono-layered, self-assembled photonic crystals (SAPCs) fabricated by using an inkjet printer exhibit extremely weak structural colors and multiple colorful holograms so that they can be utilized in anti-counterfeit measures. We demonstrate that SAPC patterns on a white background are covert under daylight, such that pattern detection can be avoided, but they become overt in a simple manner under strong illumination with smartphone flash light and/or on a black background, showing remarkable potential for anti-counterfeit techniques. Besides, we demonstrate that SAPCs yield different RGB histograms that depend on viewing angles and pattern densities, thus enhancing their cryptographic capabilities. Hence, the structural colorations designed by inkjet printers would not only produce optical holograms for the simple authentication of many items and products but also enable a high-secure anti-counterfeit technique.

The use of photonic crystals can provide promising methods for manipulating a light spectrum and its intensity^1^,^2^; thus, they have emerged as an alternative for identifying highly sophisticated counterfeits^3^,^4^,^5^. Typically, photonic crystals are fabricated as multi-layered, 3D nanostructures with diameters ranging from 150 nm to 350 nm in order to generate photonic band-gaps in which light cannot propagate through the 3D nanostructures^6^,^7^,^8^,^9^,^10^,^11^. Such multi-layered, three-dimensional (3D) nanostructures have bright colors with angle-independent spectra and are therefore always overt. However, the covert features of photonic band-gaps, if present, are necessary for hiding watermarks for practical anti-counterfeiting applications (e.g., paper bills)^12^. In fact, tunable photonic crystals can be designed and fabricated by tailoring their refractive indices, lattice constants, or spatial symmetries. However, they require somewhat complicated nanofabrication processes and rely on external stimuli, such as electrical^13^,^14^, chemical^15^,^16^,^17^,^18^,^19^, or magnetic methods^3^,^12^,^20^. Generally, engraved patterns, like two-dimensional (2D) gratings, fabricated on a mass production basis are still commonly used for holographic anti-counterfeit techniques. These grating patterns provide viewing-angle-dependent colorations but seem to be always overt. In addition, they usually exhibit the same “signature” for security^21^,^22^,^23^. For the further development of such holographic anti-counterfeiting applications, the grating patterns must not only enable covert-overt transformation similarly to tunable photonic band-gaps, but must also be unique and uncloneable.

In this work, we introduce a novel and cost-effective fabrication method of mono-layered, self-assembled photonic crystal (SAPC) patterns and report their anti-counterfeiting applications showing important features, such as covert-overt transformation and unique photonic prints. To date, top-down nanofabrication techniques, such as photo-lithography^24^,^25^, two-photon patterning^26^,^27^, and direct-writing assembly^28^,^29^, involve cost- and time-inefficient processes that employ expensive equipment because the periodicity of the photonic crystals in the visible spectrum must be of at least several hundreds of nanometers. On the other hand, bottom-up nanofabrication methods, such as self-assembly and spreading^15^,^16^,^17^, cannot produce various and complex patterns because of additive processes. For this reason, we utilized a commercially available inkjet printer for the fabrication of SAPC patterns, which has the key advantage of easy fabrication and rapid production of various photonic prints^18^.

In addition, mono-layered SAPC patterns based on the diffraction mechanism may be a promising alternative for covert-overt transformation. In principle, mono-layered 2D nanogratings designed by self-assembled nanoparticles with the same refractive index as the substrate can minimize the intensity of optical scattering, which is caused by small impedance discontinuities generated by photonic prints on the substrate. Thus, embossed patterns on a white background cannot be easily perceived by the naked eye under low-lighting conditions, thereby producing covert patterns. However, such covert patterns can easily become overt by controlling the light intensity or changing the background. Furthermore, unique photonic signatures can be obtained by manipulating imaging pixels comprising of SAPCs. Without the information of the imaging pixels, it is almost impossible to perfectly duplicate structural colorations measured optically or electrically, thus enhancing the cryptographic capability of SAPCs in a simple manner.

In this paper, we describe inkjet-printing-based fabrication methods for mono-layered SAPC patterns on various substrates with different wettabilities and the engineering of an extremely low diffracted light to produce viewing-angle-dependent structural colors. We also investigate a unique structural coloration mechanism by performing numerical simulations based on basic diffraction theory. Subsequently, we show the practical applications of the cover-overt transformation features obtained by mono-layered SAPC patterns to several items, including a chemical bottle, a paper bill, and a credit card. Finally, we demonstrate that the unique encryption of mono-layered SAPC patterns can be designed by manipulating the pattern densities of the SAPCs and the subsequent decryption of the patterns can be performed by analyzing the red, green and blue (RGB) histograms, showing remarkable potential for a sophisticated anti-counterfeit system.

## Results and Discussion

### Printing of photonic crystal patterns

**Figure 1:**
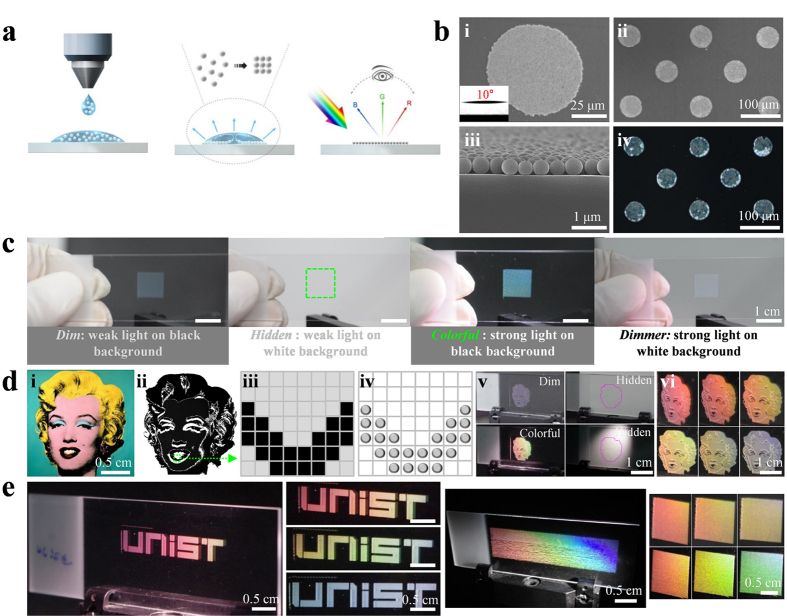
Printing, self-assembly and patterning of photonic crystals. (**a**) Illustration of the process of printing and self-assembly of photonic crystals using an inkjet printer. (**b**) SEM images of mono-layered self-assembled photonic crystal (SAPC) nanostructures (ca. 70 μm in diameter) on a glass substrate. Under strong illumination, the SAPC pattern produces colors. (**c**) The SAPC pattern (100 × 100 nanostructures per cm^2^) looks dim, hidden, colorful, or very dim, depending on the illumination intensity and background. (**d**) Structural coloration process. First, a color image (Andy Warhol Artwork © The Andy Warhol Foundation for the Visual Arts, Inc.) (i) is converted to a black-and-white one (ii). Then, it is determined on a pixel-by-pixel basis whether photonic crystals will be injected or not (iii–iv). The printed patterns can be hidden and revealed by manipulating the illumination and background conditions (v–vi). (**e**) Various images are produced by using the same structural coloration process: the word “UNIST” and a large-area pattern exhibiting rainbow colors.

A schematic of an inkjet printing process for producing various droplet patterns on a substrate is shown in Fig. 1(a). Photonic crystals (silica particles) with 500 nm diameter in injected droplets are self-assembled by drying and their nanostructural shapes and sizes are mainly determined by the surface wettability, the number density of the photonic crystal suspension, and the chemical composition of the solvents; additional characterization is displayed in Fig. S1 (Supplementary Information). For instance, Fig. 1(b) shows scanning electron microscope (SEM) images of mono-layered SAPCs on a glass substrate (i–iv). Each circular nanostructure is approximately 70 μm in diameter (i) and is comprised of dense photonic crystals (about 15,000 particles in total) (iii). An SAPC pattern is formed with a constant spacing distance of approximately 100 μm (ii) and under strong illumination, the pattern produces colors (iv). It must be noted that the photonic crystal suspension solution was mixed with formamide (FA, 20% v/v) for the close-packed and mono-layered self-assembly of the photonic crystals, the detailed mechanisms of which have been intensively studied (Note, Supplementary Information)^30^, and the number density of the photonic crystal suspension was calculated to fully fill the nanostructures and thus generate vivid structural colors (20% w/v, Fig. S2, Supplementary Information).

Here, we demonstrate that the structural colors can be overt or covert by controlling the background and the intensity of light illumination. Figure 1(c) shows an SAPC pattern on a glass substrate under weak illumination, such as office lighting (ca. 500 lux) and daylight (ca. 2,000 lux), on a black and a white background, respectively, and under strong illumination with 80-W white light (above 20,000 lux) on the same backgrounds. The photographs were obtained using a charge-coupled device (CCD) camera (iPhone 5). As shown in Fig. 1(c), under weak light illumination, the SAPC pattern is dim on the black background and concealed on the white background. This unique color visualization results from the small reflectance of the mono-layered SAPCs, which can be explained by the Bragg scattering (i.e., diffraction) that divides weak light into several rays travelling in different directions^31^. In fact, the pattern density (photonic crystal area of the substrate) was determined to cover approximately 22.1% of the substrate, resulting in low diffraction intensity (Fig. S3, Supplementary Information). Because all lights are absorbed by the black background, changing the background can manipulate the net scattering from the pattern by enhancing the signal to noise ratio (SNR). However, the SNR enhancement by the black background appears not to be sufficient to transform the covert (dim) image to an overt (vivid) one. This can be attributed to the low diffraction intensity (low reflectance). This issue can be simply resolved by employing strong illumination (e.g., smartphone flash light), which can directly enhance the diffraction intensity of the SAPC pattern. We confirm that, under strong illumination, the same pattern displays vivid colors on the black background while it looks very dim on the white background. According to the spherical coordinate system shown below, all the images were obtained when the light source and the camera were fixed at P_L_ (3 cm, 30°, −40°) and P_C_ (3 cm, 90°, 0°), respectively. Although the efficiency of the developed holograms is extremely low, because of mono-layered 2D nano-gratings, the imaging resolution can be enhanced by fabricating multi-layered nano-gratings and/or using high impedance mismatch between embossed patterns and substrates.

Next, we demonstrate an in-series fabrication process for producing structural colors in a controllable manner, as displayed in Fig. 1(d). A multi-colored image (i) must be converted to a black-and-white one (ii) so that each pixel (approximately 100 μm × 100 μm) becomes either black or white (iii). Particle suspension droplets are then printed only on the black pixels (iv). For example, we chose an image of Marilyn Monroe and then produced its photonic crystal pattern by injecting about 10,000 droplets into a 1.5 cm × 1.5 cm area. Photographs (v) of the SAPC pattern show various colorations on different backgrounds and under different illumination conditions. Similarly to Fig. 1(c), the pattern can be displayed under relatively strong illumination (80-W white light) on a black background but it is concealed or very dim under weak illumination on a white background (see Movie 1, Supplementary Information). Notably, the colorful pattern is not detected on the black background in daylight. Interestingly, clear and colorful images appear under strong illumination, and their coloration depends on the viewing angle (vi) (see Movie 2, Supplementary Information). Figure 1(e) shows representative photographs of structural colors produced using the same process as that used for Fig. 1(d), with the word “UNIST” on the left and a large-area pattern on the right. Considering that the structural colors from the SAPCs formed on a glass substrate are hidden under weak light (daylight) but are obvious under strong light, SAPCs can be directly applied to an anti-counterfeit technique.

### Effect of surface wettability on photonic crystal patterns

**Figure 2:**
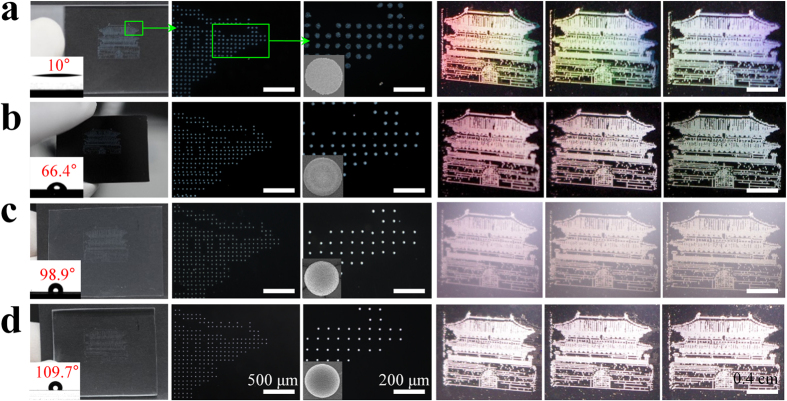
Self-assembly and patterning of photonic crystals on various substrates. (**a–d**) Comparison of the self-assembly process and the structural coloration between different substrates: (**a**) glass, (**b**) a Si wafer, (**c**) PP, and (**d**) PDMS. All the patterns (Namdaemoon © National Research Institute of Cultural Heritage, under the KOGL: Korea Open Gov. License, Type No. 1) were observed on a black background. Daylight was used as the weak light for the first three columns of images (left), whereas an 80-W white illuminator was employed as the strong light for the remaining three columns of images (right).

Figure 2 shows SAPC patterns of the “National Treasure No. 1” of Korea, called Namdaemoon; the patterns were created on various substrates with different wettability, such as glass slides, silicon wafers, polypropylene (PP), and polydimthylsiloxane (PDMS), within a 1 cm × 1.5 cm area with a 100-μm spacing distance. The inset images of Fig. 2 show the contact angles (CAs) of distilled water droplets. As the CA increases, mono-layered (glass slide, Fig. 2(a)), multi-layered (Si-wafer, Fig. 2(b)), and dome-shaped nanostructures (PP, Fig. 2(c); PDMS, Fig. 2(d)) were obtained in this order^32^. Next, photographs were acquired using white-light illumination (80 W) on the four samples. Surprisingly, the mono-layered nanostructure patterns on hydrophilic surfaces emit iridescent colors, like those of an opal, and exhibit a rainbow-like pattern at different viewing angles. On the other hand, for the dome-shaped nanostructure patterns with higher dimensional layers, colorful images no longer appear, because a photonic band-gap forms outside the visible spectrum. Notably, although many researchers have attempted to develop dome-shaped photonic crystal structures and patterns^33^,^34^, these were easily obtained in this work by using PP and PDMS without any surface treatment. It is well known that dome-shaped nanostructure patterns can be used for viewing-angle-independent display applications^6^. However, we emphasize that the mono-layered photonic crystal patterns are as useful as the multi-layered ones for displaying viewing-angle-dependent structural colorations^6^,^7^,^15^,^16^,^17^,^34^.

### Numerical calculation, measurement, and structural coloration of SAPC patterns

**Figure 3:**
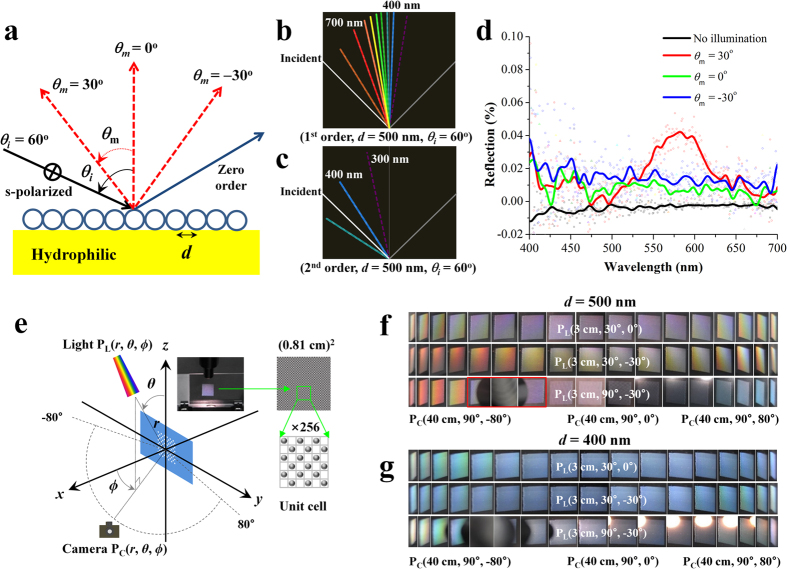
Comparison between theoretical and experimental coloration results. (**a**) A basic diffraction grating model of a mono-layered SAPC pattern with diameter *d* = 500 nm. (**b**) The first-order diffraction spectrum with an incident angle *θ*_*i*_ = 60°. (**c**) The second-order diffraction spectrum with the same incident angle as b. (**d**) Diffused reflection measurements of the SAPC pattern when an s-polarized incident wave enters at *θ*_*i*_ = 60° and a detector is placed at three different locations, *θ*_*m*_ = −30°, 0°, and 30°. (**e**) A spherical coordinate system displaying the position of the incident light P_L_(*r, θ, ϕ*) and that of the CCD camera P_C_(*r, θ, ϕ*). (**f**) Photographs of a 500-nm photonic crystal pattern acquired when the position of the incident light was located at P_L_(3 cm, 30° ~ 90°, −30° ~ 0°) and the camera was placed at P_C_(40 cm, 90°, −80° ~ 80°) on the *x*–*y* plane. (**g**) Photographs of a 400-nm photonic crystal pattern for the same light and camera positions as f.

We theoretically support the observed structural coloration with basic diffraction theory^35^,^36^. Figure 3(a) shows a grating model of SAPCs on a hydrophilic surface (mono-layered, flat structures with the particle diameter *d* = 500 nm). When rainbow spectra enter the grating element, the spectral components are diffracted into multiple colorful rays in different angular directions. The angles of the diffracted rays are related to the size of the particles and the angle of the incident light as expressed in Equation (1):


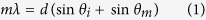


where *m* is the order of the reflected ray, *λ* is the wavelength, *θ*_*i*_ is the incident angle of the light (plane wave), and *θ*_m_ is the angle of reflection relatively to the normal plane. The first-order (*m* = 1) and second-order (*m* = 2) diffractive rays are obtained by a numerical grating calculation. In the case of an SAPC pattern with *d* = 500 nm, the first-order diffractive rays are easily detected by the naked eye (Fig. 3(b)) but high-order diffractive rays are imperceptible (Fig. 3(c)) because they are nearly in the ultraviolet spectrum. In Fig. 3(d), we quantitatively examine the structural colorations by measuring the diffused reflections from an SAPC pattern on a Si-wafer substrate. When an s-polarized incident wave (the magnetic field is perpendicular to the plane of the incident wave) enters at angle *θ*_*i*_ = 60°, diffused reflections are measured at three different points (*θ*_*m*_ = −30°, 0°, and 30°). Although the diffused reflections are very small relatively to the specular reflections (Fig. S5, Supplementary Information), they depend on the wavelength (*λ*) and the detection angle (*θ*_*m*_). For *θ*_*m*_ = 30°, the diffused reflection shows the maximum at approximately 600 nm whereas for *θ*_*m*_ = 0° and −30°, a substantial portion of the diffused reflections are located below the blue spectrum. This is in good agreement with the first-order diffraction spectra shown in Fig. 3(b). The diffused reflections also vary with the incidence angle (*θ*_*i*_), as shown in Fig. S6 (Supplementary Information).

Figure 3(e) illustrates a spherical coordinate system P(*r, θ, ϕ*) that defines the position of a light source and a CCD camera, where *r* is the distance from the origin to the light source or the CCD camera and *θ* and *ϕ* are the angles, as defined earlier. The experimental setup used for obtaining the photographs of the SAPC patterns is shown in Fig. S7 (Supplementary Information). In order to analyze the structural coloration using direct imaging, the SAPCs were placed on the *y*-*z* plane and the light source was located in different positions. Figure 3(f) shows the structural colors of the SAPC pattern (*d* = 500 nm), which was illuminated by moving the CCD camera in 10° increments from *ϕ* = −80° to *ϕ* = 80° on the *x*–*y* plane^37^. When a light source was located on the *x*-*z* plane P_L_ (3 cm, 30°, 0°), the diffractive rays produced viewing-angle-dependent colored images with symmetric patterns along the *x*-axis, which resulted from the 2D symmetric diffraction with respect to *ϕ* = 0° (see Fig. S8, Supplementary Information). On the other hand, at P_L_ (3 cm, 30°, −30°) and P_L_ (3 cm, 90°, −30°), the SAPCs exhibited asymmetrically colored images along the *x*-axis, which resulted from obliquely incident waves on the SAPCs (see Figs S9 and S10, Supplementary Information). When the light source was located at P_L_ (3 cm, 90°, −30°) at extreme angles (*ϕ* = −80° and *ϕ* = 80°), the 2D diffractive rays were monochromatic red (*ϕ* = −80°) and blue (at *ϕ* = 80°) because 2D periodic structures behave like one-dimensional gratings. We further investigated the effect of a different particle size, d = 400 nm. Figure 3(g) shows the captured images, which exhibit an almost monochromatic blue spectrum, regardless of camera angle. This can be attributed to subwavelength-periodicity elements with smaller periodicity than the visible wavelength of light, which provide mostly specular reflection (*m* = 0^th^ order) and no diffused reflections.

### Covert-overt transformation of SAPC patterns

**Figure 4:**
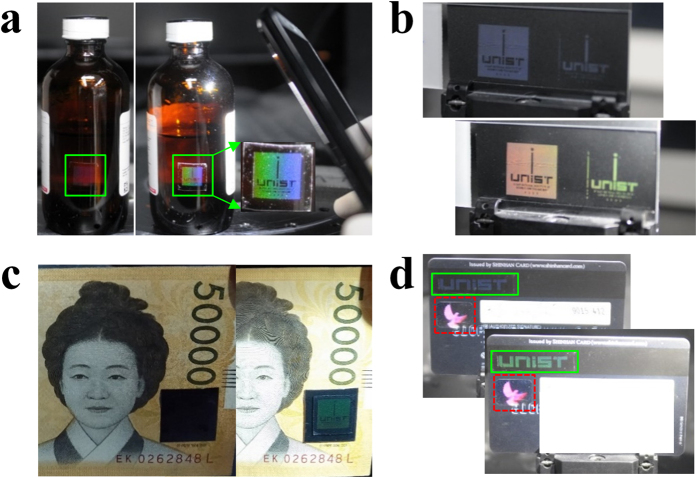
Covert-overt transformation of SAPC patterns for anti-counterfeiting applications. (**a**) An SAPC pattern of a logo is normally hidden in daylight but is revealed by additional illumination using a smartphone flash (iPhone 5). (**b**) An SAPC pattern on a PDMS slab attached to a bare glass substrate, which shows color variations that depend on viewing angles and light intensities. (**c**) An SAPC pattern on a paper bill is usually covert but is overt under a bright light. (**d**) An SAPC pattern (indicated with the green rectangle) can be overt as well as covert whereas the hologram (in the red rectangle) made by the manufacturer is always overt.

Figure 4 shows a simple bi-directional transformation between covert and overt patterns, achieved by manipulating the background and/or illumination intensities. First, we produced a pattern on an oxygen-plasma-treated PDMS slab with hydrophilic wettability and flexibility (CA = 10° and thickness 200 μm), whose surfaces were well-attached to the glass surfaces^38^. Interestingly, the pattern could be concealed on a conventional chemical glass bottle but became detectable with a smartphone camera flash or light (Fig. 4(a)). Figure 4(b) shows that the same pattern fabricated on a bare glass substrate did not exhibit a colorful image under daylight, but did so under strong light illumination (see Movie 3, Supplementary Information). A more realistic anti-counterfeiting application was demonstrated with a paper bill (Fig. 4(c)) and a credit card (Fig. 4(d)), on which clear images were obtained under strong illumination and on a black background. Thus, concealed photonic prints can be obtained by controlling the light density and background color. Notably, the covert-overt transformation feature of photonic crystal patterns has never been reported in the previous literature. In particular, mono-layered nanostructure patterns based on 2D nanogratings have been attracting less attention than multi-layered ones (photonic band-gaps) although they can be very useful in visual judgement-based anti-counterfeiting applications. The fact that SAPC patterns are not stable on substrates may cause a problem in practical applications; however, this problem was easily resolved by attaching an equally sized bare PDMS slab on top of the SAPC patterns. As a result, the patterns were sandwiched for permanent anti-counterfeit use. This arrangement was proven to be robust for long-term use.

### RGB histogram of SAPC patterns

**Figure 5:**
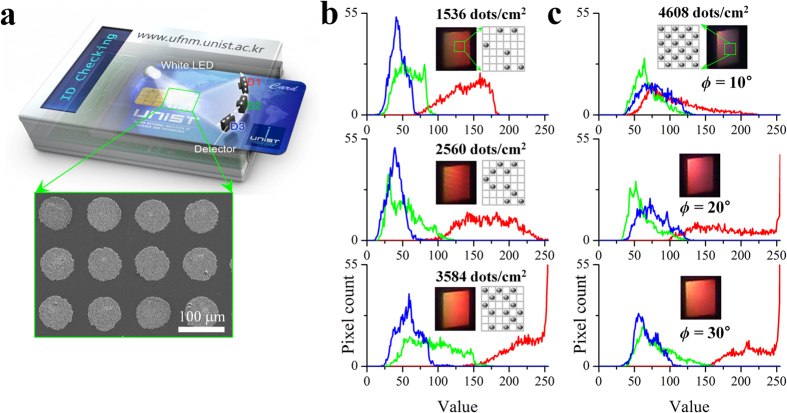
RGB histograms of SAPC patterns for an anti-counterfeit system. (**a**) A conceptual anti-counterfeit system for a credit card, consisting of a light source (white LED) and three detectors (D1, D2, and D3). (**b**) 8-bit CCD images of 1 cm × 1 cm SAPC patterns with different pattern densities (6, 10, and 14 out of 36 unit dots) were obtained by a regular smartphone at P_C_(40 cm, 90°, 30°) and then separated into red, green, and blue histograms, respectively. Different pattern densities exhibit different RGB histograms. (**c**) The histograms of 8-bit CCD images acquired at different viewing angles, such as *ϕ* = 10°, 20°, and 30° (i.e., P_C_(40 cm, 90°, 10° ~ 30°)), show significant profile shifts.

Figure 5(a) shows a conceptual authentication system that consists of a light source (white LED) and multiple-light detectors (inexpensive CCD cameras), which is used to measure the structural colors of an SAPC pattern at different viewing points. The SAPC pattern can be optically quantified by analyzing the RGB histograms of the structural colorations. In order to assign a unique identity to the SAPC pattern, we modulated the pattern densities of the imaging pixels. Figure 5(b) shows the RGB histograms of three SAPC patterns with different pattern densities, as shown in the insets. Namely, each unit cell had 36 nanostructures (pixels) in a 6 × 6 matrix format. For example, only 6, 10, and 14 out of 36 pixels were printed and then tested, corresponding to 1536, 2560, and 3584 dots/cm^2^, respectively. Images were obtained by a regular smartphone (iPhone 5) and showed unique structural colorations^39^. As expected, the profiles of the RGB histograms moved to higher wavelengths as the number density of the SAPC patterns increased because of the increase in intensity. In addition, when the viewing angle (camera position) changed, as shown in Fig. 5(c), we observed a profile shift of the RGB histograms, thus ensuring the realization of a highly sensitive anti-counterfeit system with high resolution. Namely, greater |*ϕ*| values produced greater shifts of the RGB histograms. Notably, this shows good agreement with Figs S8–S10 (Supplementary Information). In this context, we concluded that the pattern density and the viewing angle dependency increase the cryptographic capabilities of SAPC patterns, and the multiple use of light sources and detectors enhances the security and reliability of the anti-counterfeit capacity.

Finally, we assessed the repeatability of SAPC patterns. As shown in Fig. S11, three SAPC patterns were produced as identically as possible by using the exactly same printing conditions but they resulted in microscopically and nanoscopically different patterns. Not only the perimeter of SAPC nanostructures was different from each other but also the centre-to-centre alignment was slightly distorted. This seems to be caused by the systematic uncertainties related to different droplet evaporation conditions, various droplet flight trajectories from the nozzle to the substrate, locally non-uniform surface morphologies and wettabilities, and so on during the printing and self-assembly. However, the patterns showed almost the same RGB histograms as shown in Fig. S12. Therefore, we confirmed that the inkjet-printing method can guarantee the sufficient fabrication repeatability of SAPC patterns for practical anti-counterfeiting applications.

## Conclusion

In conclusion, we demonstrated a new class of anti-counterfeit techniques using 2D nanograting patterns that were artificially designed and produced by inkjet printers. We confirmed the bi-directional, covert-overt transformation of photonic crystal patterns in a very simple manner, by using smartphone light and a black background. Furthermore, we established the capability of 2D nanogratings to achieve optical encryption and decryption using the RGB histograms of colorful images obtained by a regular smartphone. Lastly, we emphasize several unique features that distinguish the proposed technique from other alternatives: facile fabrication of various 2D nanogratings (simplicity, cost-effectiveness, and convenience), extremely low-reflectance engineering (covert), holographic anti-counterfeiting with low security based on covert-overt transformation, and optical encryption/decryption of unique SAPC patterns for sophisticated anti-counterfeit systems.

## Methods

### Reagent and materials

Monodisperse plain silica particles (10% w/v) with diameters of 400 nm and 500 nm (Polysciences, Warrington, USA) were used. To manipulate the final working concentration of the particles, the following centrifugal steps were employed. First, a 1000 μL solution of silica particles as delivered was centrifuged at 4000 rpm for 12 minutes, and then the solution was removed for a high concentration particle suspension (>20% w/v) or diluted with deionized water for a low concentration particle suspension (<10% w/v). Then, the particle suspension was mixed with FA (Sigma Aldrich, Korea) for the final concentration to be 20% v/v to control evaporation time for better self-assembly^33^. The prepared, mixed particle suspension was treated ultrasonically for 10 minutes for complete dispersion (5510E-DTH, Bransonic, USA). In parallel, four different substrates were prepared to investigate effects of hydrophobicity and hydrophilicity of substrates on the crystallization of nanobeads such as slide glass, Si-wafer, PDMS, or PP. Finally, all substrates were carefully cleaned in acetone and IPA, rinsed with deionized water, and dried with a stream of N_2_. No further surface modifications were made.

### Materials printer

We used a piezoelectric drop-on-demand inkjet printer manufactured by Fujifilm Dimatix, Inc. (DMP-2800, CA, USA) with a cartridge (Model No. DMC-11610) that supports 10 pL droplets. These were used in all of the experiments. The printer head consists of 16 nozzles in a row with a 254 μm spacing distance. Each nozzle is approximately 21.5 μm in diameter and their operation can be controlled individually. The center-to-center drop spacing is adjustable in one-micron increments within a 5 μm to 254 μm range and is dependent upon the dpi setting. The 5 μm drop spacing corresponds to a 5080 dpi maximum printing resolution while 254 μm corresponds to a 100 dpi minimum printing resolution. Mainly 254 dpi corresponding to a drop spacing of 100 μm was used because it kept an appropriate distance to prevent overlapping between droplets^40^.

### Spectrum measurement

Spectra were measured by using a spectrophotometer (Cary 5000 UV-vis-NIR, Santa Clara, CA, USA) in order to quantitatively measure diffractive rays. Figure S5(a) (Supplementary Information) shows the experimental setup used to obtain diffused and specular reflections. In this experiment, we used an SAPC pattern with diameters of 500 nm on a Si-wafer substrate. A diffraction grating model of a mono-layered photonic crystal pattern is shown in Fig. S5(b) (Supplementary Information) and the comparison between diffused (*θ*_*m*_ = 0°) reflection and specular reflection (*θ*_*m*_ = 60°) is displayed in Fig. S5(c) (Supplementary Information) when an s-polarized incident wave enters at *θ*_*i*_ = 60°. As expected, the specular reflection occupies the greater part of the net reflection. Figure S5 (Supplementary Information) shows the measured diffracted spectra of an s-polarized incident wave with an incidence angle of *θ*_*i*_ = 45° and 30°, respectively. In these cases, most diffused reflections appear at the blue light of the visible spectrum.

### Experimental setup and data analysis

The CA of various substrates was measured by gently dropping deionized water droplets of 2 μL onto the various substrates and then using a goniometer equipped with an optical system and a CCD camera (Model 200, Ramé-Hart Instrument Co., Succasunna, NJ, USA) to perform measurements. All the CAs reported in this study have been averaged from at least 10 measurements. Scanning electron microscope (SEM) images were taken and utilized to study the morphology and spatial distribution of the resulting silica nanostructures/deposits (S-4800, Hitachi, Japan). Micrographs were taken by using a high-resolution CCD camera (Eclipse 80i, Nikon, Japan). Two types of CCD cameras were used: an SLR CCD camera (Nikon D300, Nikon, Japan) for high quality images and an iPhone 5 for regular images. Camera positions were carefully manipulated by using a custom zig setup as shown in Fig. S6 (Supplementary Information) to obtain images from different viewing angles. All microscopic images were processed and quantified using Image J (NIH, Bethesda, MD, USA) and graphs were drawn using OriginPro 8 (OriginLab, Wheeling, IL, USA).

## Additional Information

**How to cite this article**: Nam, H. *et al*. Inkjet Printing Based Mono-layered Photonic Crystal Patterning for Anti-counterfeiting Structural Colors. *Sci. Rep.* **6**, 30885; doi: 10.1038/srep30885 (2016).

## Supplementary Material

Supplementary Information

Supplementary Information

Supplementary Information

Supplementary Information

## References

[b1] Yablonovitch E. Inhibited spontaneous emission in solid-state physics and electronics. Phys. Rev. Lett. 58, 2059 (1987). 10034639 10.1103/PhysRevLett.58.2059

[b2] John S. Strong localization of photons in certain disordered dielectric superlattices. Phys. Rev. Lett. 58, 2486 (1987). 10034761 10.1103/PhysRevLett.58.2486

[b3] Hu H., Chen Q.-W., Tang J., Hu X.-Y. & Zhou X.-H. Photonic anti-counterfeiting using structural colors derived from magnetic-responsive photonic crystals with double photonic bandgap heterostructures. J. Mater. Chem. 22, 11048–11053 (2012).

[b4] Han S. . Lithographically Encoded Polymer Microtaggant Using High‐Capacity and Error‐Correctable QR Code for Anti‐Counterfeiting of Drugs. Adv. Mater. 24, 5924–5929 (2012). 22930454 10.1002/adma.201201486

[b5] Chen L. . The temperature‐sensitive luminescence of (Y, Gd) VO4: Bi3+, Eu3+ and its application for stealth anti‐counterfeiting. Phys. Status Solidi Rapid Res. Lett. 6, 321–323 (2012).

[b6] Kuang M. . Inkjet Printing Patterned Photonic Crystal Domes for Wide Viewing-Angle Displays by Controlling the Sliding Three Phase Contact Line. Adv. Opt. Mater. 2, 34–38 (2014).

[b7] Wang J., Wang L., Song Y. & Jiang L. Patterned photonic crystals fabricated by inkjet printing. J. Mater. Chem. C 1, 6048–6058 (2013).

[b8] Cui L. . Fabrication of large-area patterned photonic crystals by ink-jet printing. J. Mater. Chem. 19, 5499–5502 (2009).

[b9] Kang P., Ogunbo S. O. & Erickson D. High Resolution Reversible Color Images on Photonic Crystal Substrates. Langmuir 27, 9676–9680 (2011). 21766808 10.1021/la201973b

[b10] Gu Z.-Z., Fujishima A. & Sato O. Fabrication of High-Quality Opal Films with Controllable Thickness. Chem. Mater. 14, 760–765 (2002).

[b11] Wong S., Kitaev V. & Ozin G. A. Colloidal Crystal Films: Advances in Universality and Perfection. J. Am. Chem. Soc. 125, 15589–15598 (2003). 14664606 10.1021/ja0379969

[b12] Hu H. . Invisible photonic printing: computer designing graphics, UV printing and shown by a magnetic field. Sci. Rep. 3, (2013). 10.1038/srep01484 PMC3601367 23508071

[b13] Lu Y., Xia H., Zhang G. & Wu C. Electrically tunable block copolymer photonic crystals with a full color display. J. Mater. Chem. 19, 5952–5955 (2009).

[b14] Arsenault A. C., Puzzo D. P., Manners I. & Ozin G. A. Photonic-crystal full-colour displays. Nature Photon. 1, 468–472 (2007).

[b15] Zhang J. T., Wang L. L., Chao X. & Asher S. A. Periodicity-Controlled Two-Dimensional Crystalline Colloidal Arrays. Langmuir 27, 15230–15235 (2011). 22007604 10.1021/la203363e

[b16] Zhang J. T., Wang L. L., Lamont D. N., Velankar S. S. & Asher S. A. Fabrication of Large-Area Two-Dimensional Colloidal Crystals. Angew. Chem. Int. Ed. 51, 6117–6120 (2012). 10.1002/anie.201105439 22566073

[b17] Zhang J. T., Wang L. L., Chao X., Velankar S. S. & Asher S. A. Vertical spreading of two-dimensional crystalline colloidal arrays. J. Mater. Chem. C 1, 6099–6102 (2013).

[b18] Bai L. . Bio-Inspired Vapor-Responsive Colloidal Photonic Crystal Patterns by Inkjet Printing. ACS Nano 8, 11094–11100 (2014). 25300045 10.1021/nn504659p

[b19] Ge J. & Yin Y. Responsive photonic crystals. Angew. Chem. Int. Ed. 50, 1492–1522 (2011). 10.1002/anie.200907091 21254316

[b20] Ge J., Hu Y. & Yin Y. Highly Tunable Superparamagnetic Colloidal Photonic Crystals. Angew. Chem. Int. Ed. 46, 7428–7431 (2007). 10.1002/anie.200701992 17610234

[b21] Lee R. A. Micro-technology for anti-counterfeiting. Microelectron Eng 53, 513–516 (2000).

[b22] Koivukunnas P. & Kosonen H. Invetors; Avantone Oy, assignee. Anti-counterfeit hologram. United States Patent US **8**,105,677. (31 Jan 2012).

[b23] Blyth J. Inventor; Blyth J, assignee. Hologram identification device. United States Patent US 4,563,024. (7 Jan 1986).

[b24] Campbell M., Sharp D., Harrison M., Denning R. & Turberfield A. Fabrication of photonic crystals for the visible spectrum by holographic lithography. Nature 404, 53–56 (2000). 10716437 10.1038/35003523

[b25] Jeon S., Malyarchuk V., Rogers J. A. & Wiederrecht G. P. Fabricating three-dimensional nanostructures using two photon lithography in a single exposure step. Opt. Express 14, 2300–2308 (2006). 19503567 10.1364/oe.14.002300

[b26] Cumpston B. H. . Two-photon polymerization initiators for three-dimensional optical data storage and microfabrication. Nature 398, 51–54 (1999).

[b27] Kawata S., Sun H.-B., Tanaka T. & Takada K. Finer features for functional microdevices. Nature 412, 697–698 (2001). 11507627 10.1038/35089130

[b28] Deubel M. . Direct laser writing of three-dimensional photonic-crystal templates for telecommunications. Nat. Mater. 3, 444–447 (2004). 15195083 10.1038/nmat1155

[b29] Gratson G. M., Xu M. & Lewis J. A. Microperiodic structures: Direct writing of three-dimensional webs. Nature 428, 386–386 (2004). 15042080 10.1038/428386a

[b30] Park J. & Moon J. Control of colloidal particle deposit patterns within picoliter droplets ejected by ink-jet printing. Langmuir 22, 3506–3513 (2006). 16584221 10.1021/la053450j

[b31] Born MaW E. Principles of Optics: Electromagnetic Theory of Propagation, Interference,and Diffraction of Light. CUP Archive (1966).

[b32] Li Y., Wang F., Liu H. & Wu H. Nanoparticle-tuned spreading behavior of nanofluid droplets on the solid substrate. Microfluid Nanofluid 1–10 (2015).

[b33] Park J., Moon J., Shin H., Wang D. & Park M. Direct-write fabrication of colloidal photonic crystal microarrays by ink-jet printing. J. Colloid Interface Sci. 298, 713–719 (2006). 16458916 10.1016/j.jcis.2006.01.031

[b34] Wang L. . Inkjet printed colloidal photonic crystal microdot with fast response induced by hydrophobic transition of poly (N-isopropyl acrylamide). J. Mater. Chem. 22, 21405–21411 (2012).

[b35] Yeh S. L. & Lin S. T. Identifying a dot-matrix hologram by checking the intersecting angles of its gratings. Opt. Commun. 283, 243–248 (2010).

[b36] Yao J. . Selective appearance of several laser-induced periodic surface structure patterns on a metal surface using structural colors produced by femtosecond laser pulses. Appl Surf Sci 258, 7625–7632 (2012).

[b37] Tang M. . Angle coder of anti-counterfeiting color in optical micro-mirror arrays. Optik 124, 6146–6148 (2013).

[b38] Bhattacharya S., Datta A., Berg J. M. & Gangopadhyay S. Studies on surface wettability of poly (dimethyl) siloxane (PDMS) and glass under oxygen-plasma treatment and correlation with bond strength. J Microelectromech Syst 14, 590–597 (2005).

[b39] Ecker M. & Pretsch T. Multifunctional poly (ester urethane) laminates with encoded information. RSC Adv. 4, 286–292 (2014).

[b40] Choi W. S., Ha D., Park S. & Kim T. Synthetic multicellular cell-to-cell communication in inkjet printed bacterial cell systems. Biomaterials 32, 2500–2507 (2011). 21208654 10.1016/j.biomaterials.2010.12.014

