# Supplementary material for: Inkjet Printing Based Mono-layered Photonic Crystal Patterning for Anti-counterfeiting Structural Colors
Source: Sci Rep. 2016 Aug 4;6:30885. doi: 10.1038/srep30885 (PMC4973252; doi:10.1038/srep30885)
Supplement: Supplementary Information [file srep30885-s1.pdf]

# Supplementary Information for

# Inkjet Printing Based Mono-layered Photonic Crystal Patterning for Anti-counterfeiting Structural Colors

*Hyunmoon Nam* [§,†], *Kyungjun Song* [⊥,†], *Dogyeong Ha* [§], *and Taesung Kim* [§,*]

[§] Department of Mechanical Engineering, Ulsan National Institute of Science and Technology (UNIST), 50 UNIST-gil, Eonyang-eup, Ulsan 689-798, Republic of Korea.

[⊥] Korea Institute of Machinery and Materials, 156, Gajeongbuk-Ro, Yuseong-Gu, Daejeon 305-343, Republic of Korea.

Correspondence: Taesung Kim (email: tskim@unist.ac.kr)

## Supplementary Note

### Behaviour of a particle suspension droplet on the substrate

Fig. S1 shows the basic mechanism for producing photonic crystal patterns on a hydrophilic or hydrophobic substrate using an inkjet printing process. Particles with a diameter of 500 nm in a droplet are self-assembled on various substrates and then form photonic crystals during an evaporation process. First, we observed photonic crystal self-assembly on a hydrophilic glass substrate with a contact angle of 10° (Fig. S1(a)). Particles were suspended in pure water, and the final densities were 10% (w/v) and 20% (w/v), respectively. Both of the densities produced a multi-layered, coffee-ring shape pattern as shown in Fig. S1(d). Since a droplet spreads widely (high wettability) evaporation occurs more actively at the edge than at the centre. Temperature differences cause not only unbalanced surface tensions along the water-air interface, but also fluid flow within the droplet, as illustrated with arrows. The induced fluid flow replenishes fluid at the edge so that the particles are also transported to the edge[1]. This is called Marangoni flow, which produces a coffee-ring-shaped pattern in which several layers form along the perimeter.

Second, particle suspensions were blended with formamide (FA) of which final concentration was 20% v/v. However, the final densities of particles remained unchanged as used before (10% w/v and 20% w/v). In this case, the low density suspension (10% w/v) formed a mono-layered, coffee-ring-shaped pattern because of the low number density of particles in a droplet (not shown), while the highly concentrated suspension (20% w/v) produced a flat, mono-layered pattern (Fig. S1(b)). The high particle density was theoretically predicted to produce a flat, mono-layered photonic crystal structure on a hydrophilic substrate via the calculation of the particle number that could fully fill a circle, as shown in Fig. S1(e). The ideal concentration of the solvent (FA) was experimentally determined. We note that the solvent concentration (water/FA mixture) plays a more crucial role than the number density of particles in forming a flat, mono-layered self-assembly when we control the

evaporation rate. This is because FA has a higher boiling point than water while the former has lower surface tensions than the latter as reported in other literature[2, 3].

Third, the high density suspension (20% w/v) was injected onto a hydrophobic polydimethylsiloxane (PDMS) substrate as shown in Fig. S1(c), and we observed that the particles formed a dome-shaped, multi-layered nanostructure (Fig. S1(f)). Interestingly, the size of the nanostructure, but not the shape of it, was much affected by the particle density and the solvent concentration. The hydrophobic surface appears to make the contact area of a droplet shrink, unlike the hydrophilic surface, and the shape of the droplet semi-spherical. Since evaporation takes place more vigorously at the centre than at the outer edge, the self-assembly of the particles becomes dome-shaped, which is distinctly different from the hydrophilic surface[4].

## Supplementary Figures

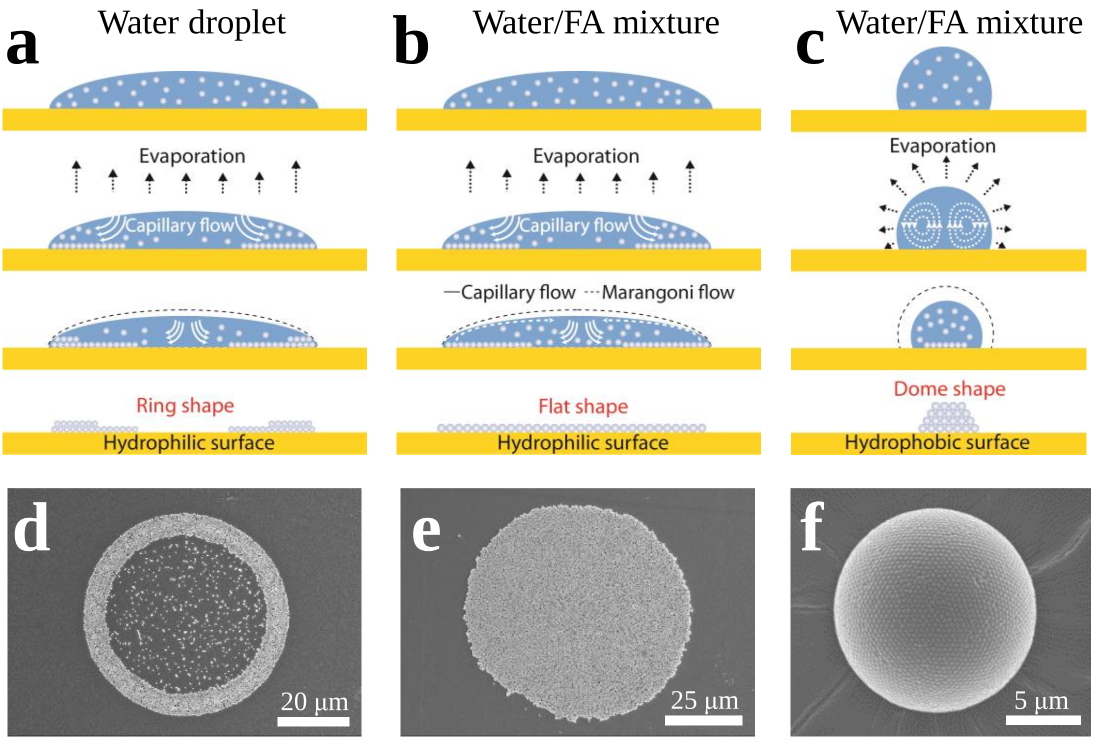


**Figure S1.** **a–c**, Schematic illustrations of evaporation and self-assembly process in a droplet. Photonic crystals are self-assembled and form different photonic crystal nanostructures on hydrophilic and hydrophobic surfaces. **d–f**, SEM images show (d) a coffee-ring-shaped, multi-layered nanostructure, (e) a circular, mono-layered one, and (f) a dome-shaped, multi-layered one.

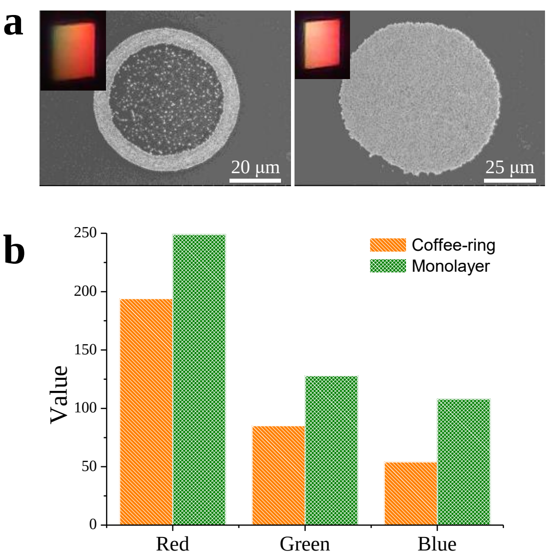


**Figure S2.** Reflectance (diffraction intensity) can be additionally manipulated by controlling the number density of photonic crystal suspension (droplets). **a**, **b**, Coffee-ring nanostructures emit weaker diffraction rays than fully-filled, monolayer ones.

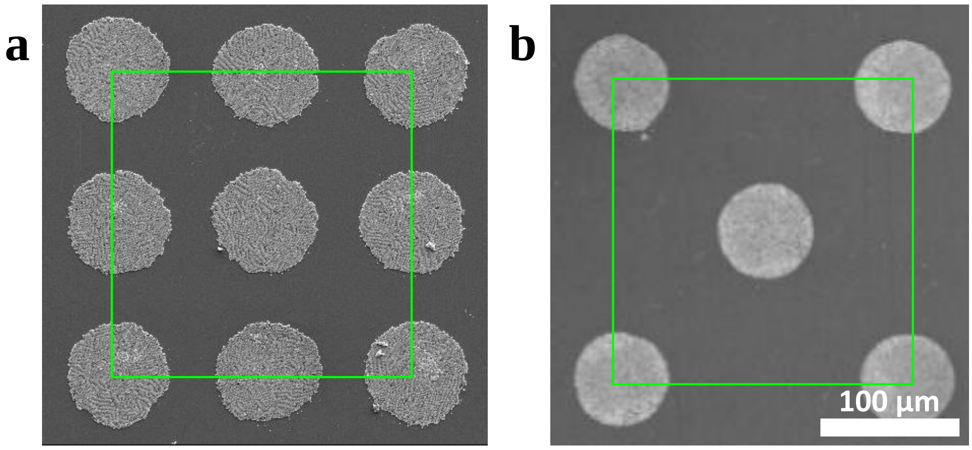


**Figure S3**. Low reflectance can be engineered by adjusting the pattern density. **a**, The maximal pattern density is achieved by injecting droplets with a spacing distance of 100 μm in a matrix format so that the patterned area covers about 44.2% out of the bare substrate surface. The unit substrate area indicated with a green square has four circular nanostructures. **b**, The pattern density is reduced by half by manipulating the number of droplets from four to two in the same unit area. As a result, the patterned area covers about 22.1%, showing how to engineer the net reflectance to hide the pattern.

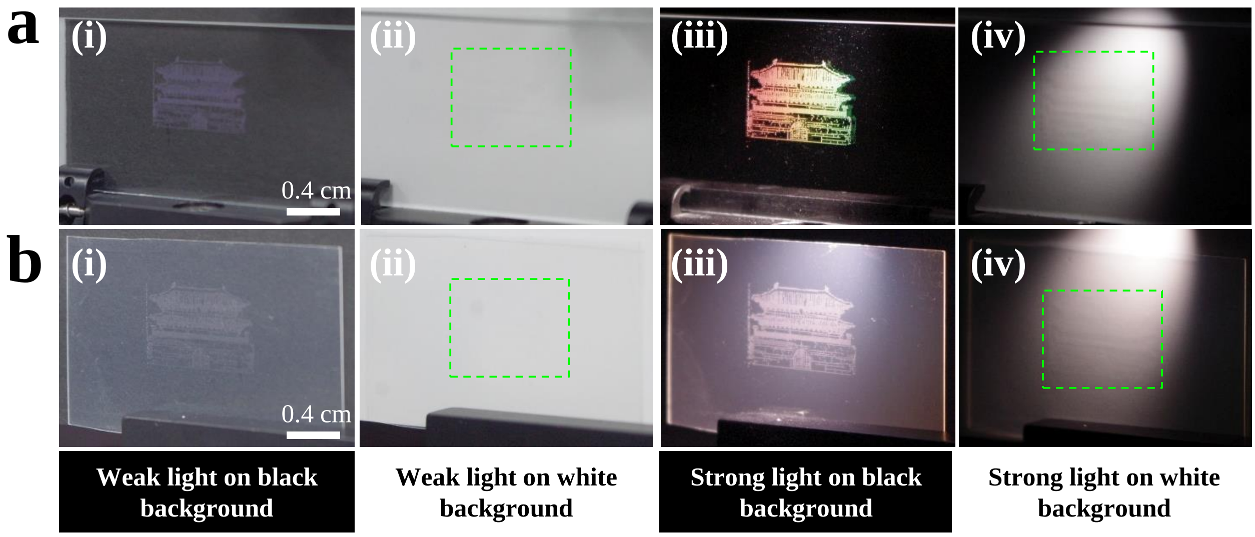


**Figure S4. a**, A flat, mono-layered SAPC pattern on the glass substrate can be seen in daylight on a black background (i). The pattern shows colours under a strong illumination on a black background (iii), and its iridescent colouration depends on the illumination intensity and the viewing angle. Interestingly, the pattern can be hidden on a white background, regardless of the illumination intensity (ii and iv). **b**, A dome-shaped, multi-layered SAPC pattern can be seen as whitish on a black background (i and iii), while it can be hidden or is very dim on a white background (ii and iv) regardless of the illumination intensity. The original image is copyrighted: Namdaemoon © National Research Institute of Cultural Heritage, under the KOGL: Korea Open Gov. License, Type No. 1.

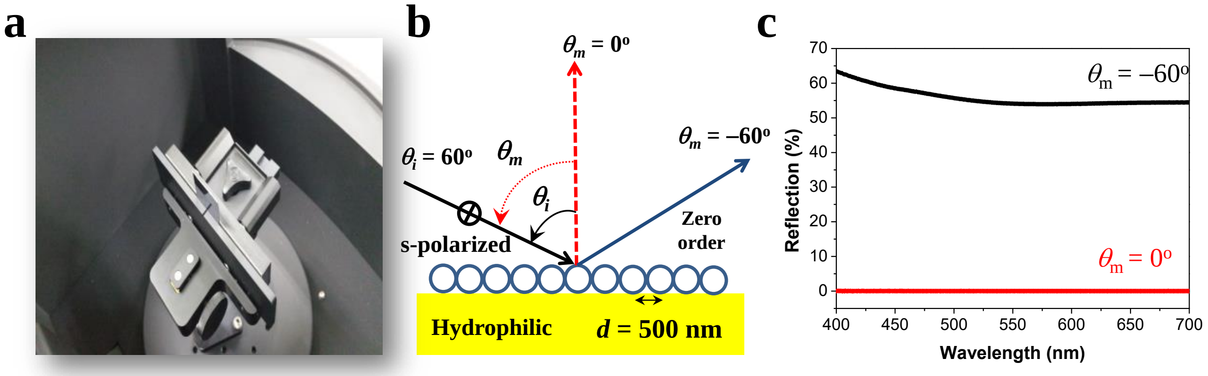


**Figure S5. a**, Experimental setup for reflection measurement. **b**, A diffraction grating model of a mono-layered SAPC with $d$ = 500 nm. **c**, Diffused ($\theta_m$ = 0°) and specular ($\theta_m$ = -60°) reflection measurement of the SAPC pattern when an s-polarized incident wave enters at $\theta_i$ = 60°. The same pattern was used in this experiment as shown in Figs. 1b,c.

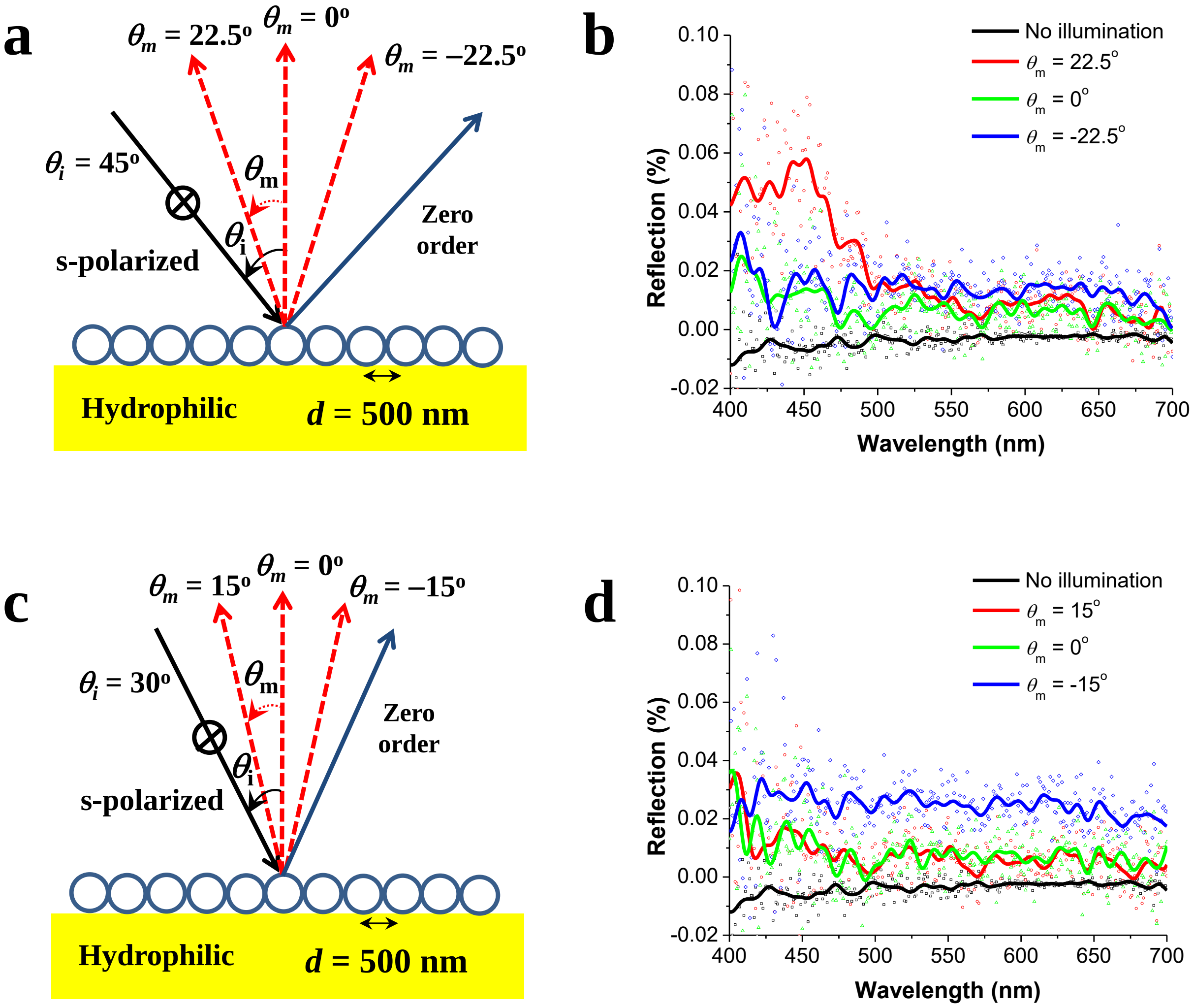


**Figure S6.** **a**, A diffraction grating model of a mono-layered SAPC (*d* = 500 nm). **b**, Diffused reflection measurements of the SAPC pattern as shown in Figs. 1b,c when an s-polarized incident wave enters at $\theta_i$ = 45° and a detector is placed at three different locations $\theta_m$ = -22.5°, 0°, and 22.5°. **c**, A diffraction grating model of a mono-layered SAPC pattern (*d* = 500 nm). **d**, Diffused reflection measurements of the SAPC pattern when an s-polarized incident wave enters at $\theta_i$ = 30° and a detector is placed at three different locations $\theta_m$ = -15°, 0°, and 15°. For both graphs, the control data were obtained in the absence of illumination from a bare Si-wafer substrate (No illumination). The same pattern was used in this experiment as shown in Figs. 1b,c.

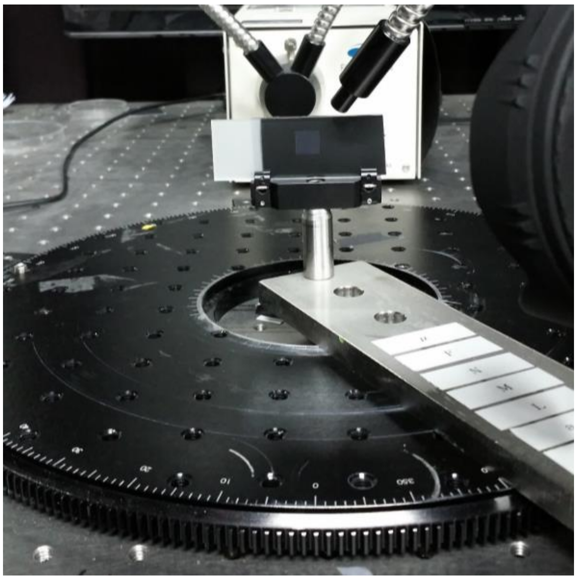

**Figure S7.** Experimental setup with a light source and a camera. The camera position was carefully manipulated by using an optical turn table that enabled us to take photographs from a wide range of viewing angles.

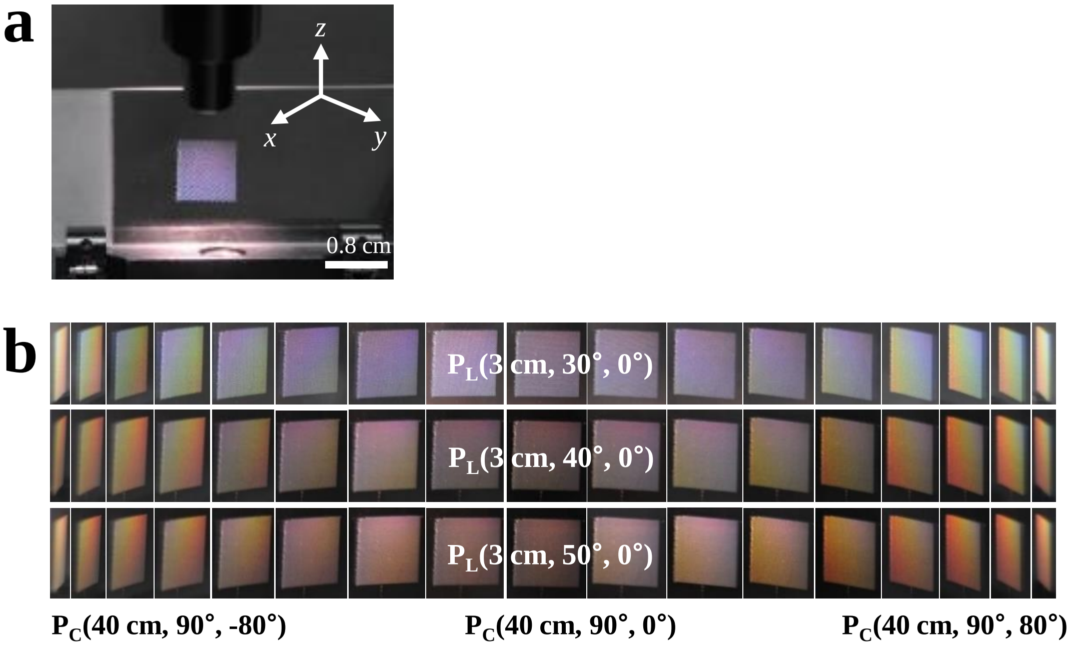


**Figure S8**. **a**, An incident light was fixed at three different locations $\theta$ = 30°, 40° and 50°, (i.e., $P_L$(3 cm, 30° ~ 50°, 0°), and a CCD camera turned on the *x*-*y* plane at 10° intervals from $\phi$ = -80° to $\phi$ = 80° (i.e., $P_C$(40 cm, 90°, -80 ~ 80°)). **b**, Photographs show colour variation as the camera location (viewing angle) changes. The colour variation is symmetrical with respect to $\phi$ = 0°. The particles are 500 nm in diameter.

**a**

**b**

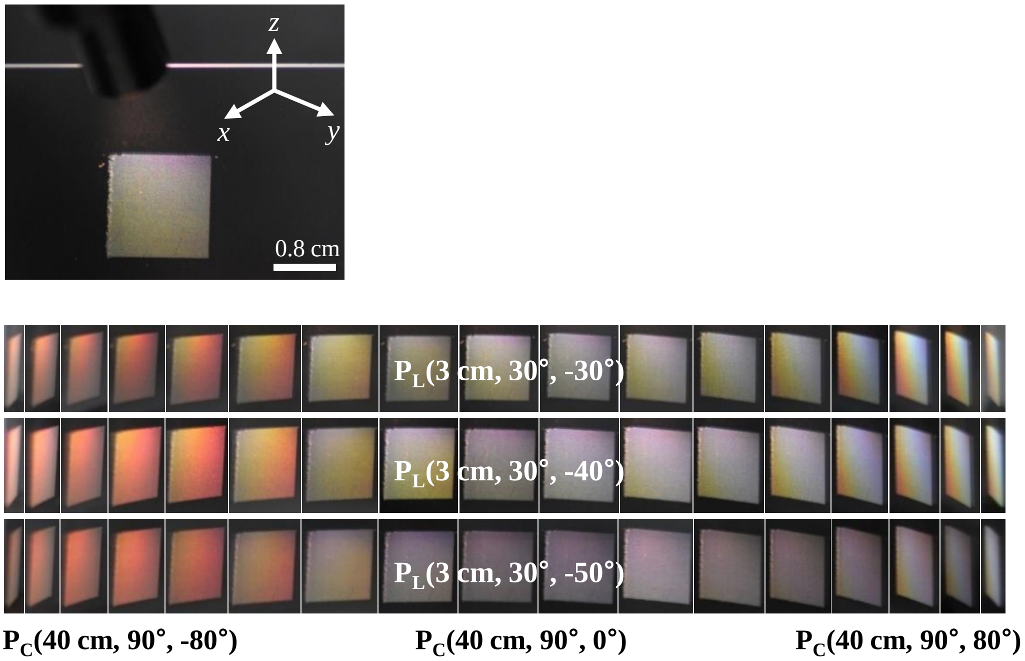


**Figure S9. a**, Incident light was fixed at three different locations $\phi$ = -30°, -40° and -50°, (i.e., $P_L$(3 cm, 30°, -30 ~ -50°), and a CCD camera turned on the *x*-*y* plane at 10° intervals from $\phi$ = -80° to $\phi$ = 80° (i.e., $P_C$(40 cm, 90°, -80 ~ 80°). **b**, Photographs show colour variation as the camera location (viewing angle) changes. The colour variation is asymmetrical with respect to $\phi$ = 0°. The particles are 500 nm in diameter.

a

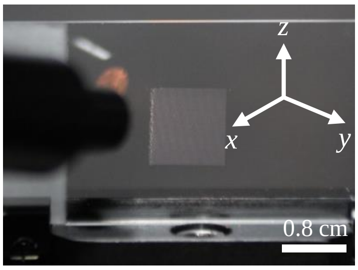


b

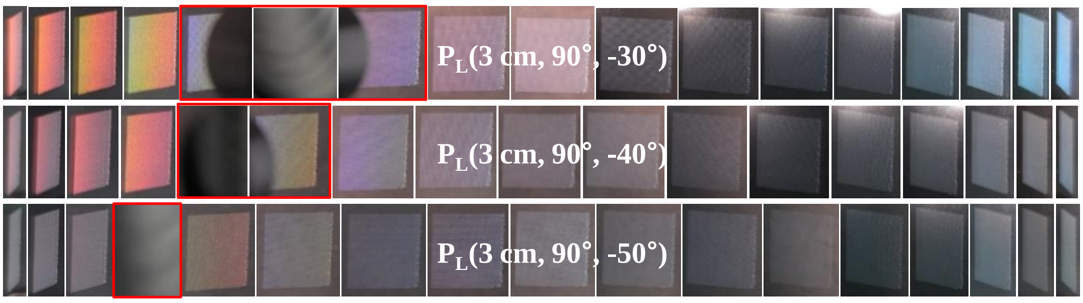


**Figure S10. a**, Incident light was fixed at three different locations $\phi$ = -30°, -40°, and -50° (i.e., $P_L$(3 cm, 30°, -30 ~ -50°)), and a CCD camera turned on the *x*-*y* plane at 10° intervals from $\phi$ = -80° to $\phi$ = 80° (i.e., $P_C$(40 cm, 90°, -80 ~ 80°)). **b**, Photographs show colour variations as the camera location (viewing angle) changes. The colour variation is asymmetrical with respect to $\phi$ = 0° because of the fixed incident light. The particles are 500 nm in diameter.

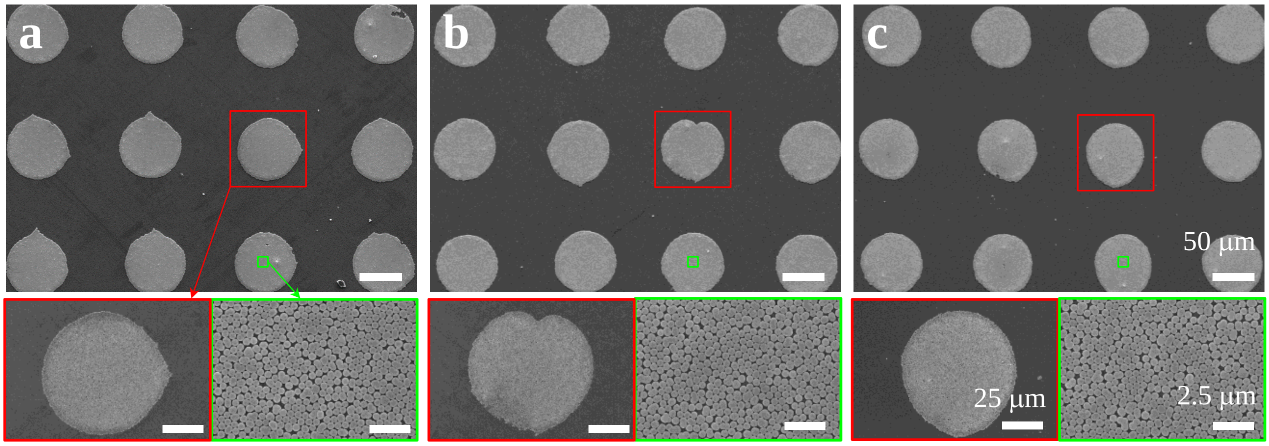


**Figure S11.** a)–c) Three SAPC patterns were produced as identically as possible by using the exactly same printing conditions such as the same bitmap image file, inkjet printer, and particle ink but resulted in microscopically (red rectangles) and nanoscopically (green rectangles) different patterns.

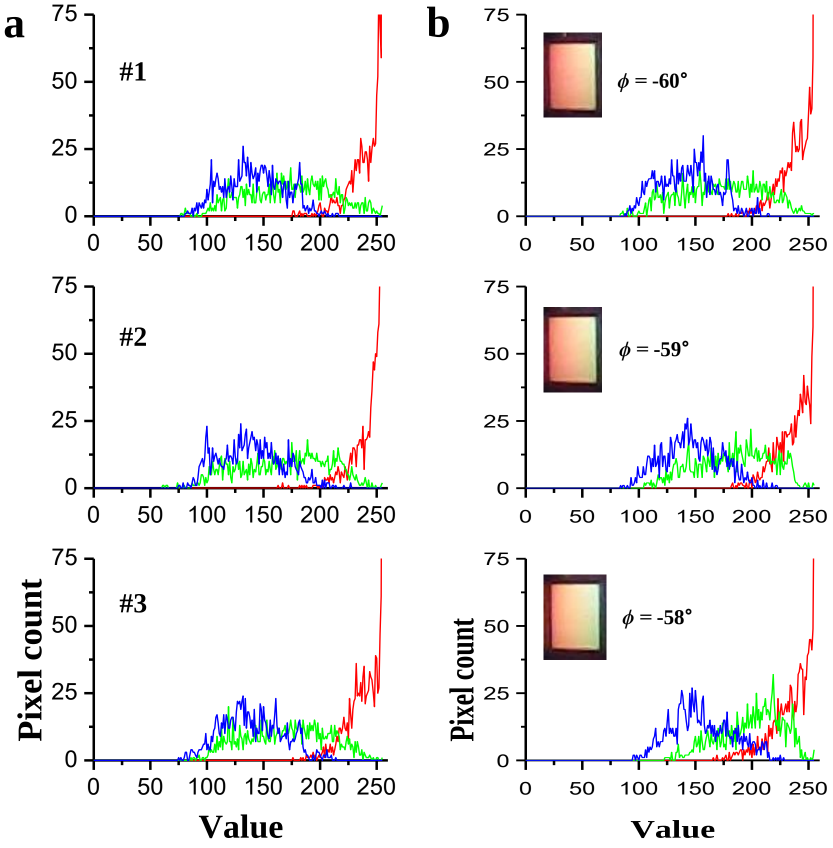


**Figure S12.** a) Three colorful images were obtained from the three SAPC patterns produced in Figure S11 separately by using the exactly same imaging conditions. And then, the RGB histograms of the images were analyzed and then drawn, showing slightly different profiles of pixel intensities. Even though the patterns were not identical at the microscale and nanoscale, the RGB histograms are almost identical. Therefore, the SAPC patterns can be optically decrypted in a simple manner using a light source and a light detector located at $P_L$(3 cm, 30°, -40°) and $P_C$(40 cm, 90°, -60°), respectively. b) The RGB histograms of the #1 SAPC pattern were analyzed from the colorful images taken at three different camera positions (i.e. $P_C$(40 cm, 90°, -58° ~ -60°) as shown in the insets. The profile shift turned out to be very sensitive to the viewing angles, demonstrating the feasibility for a high-secure anti-counterfeit system. The light source was fixed at $P_L$(3 cm, 30°, -40°).

## Supplementary Movies

**Movie 1.** Covert-overt transformation of mono-layered self-assembled photonic crystal patterns under four different experimental conditions such as weak light illumination on a black background, strong light illumination on a black background, strong light illumination without a background, and strong light illumination on a white background.

**Movie 2.** Mono-layered self-assembled photonic crystal patterns exhibit colourful structural coloration that depends on the viewing angle.

**Movie 3.** Practical anti-counterfeit applications of mono-layered self-assembled photonic crystal patterns.

## References


1. Deegan RD, Bakajin O, Dupont TF, Huber G, Nagel SR, Witten TA. Capillary flow as the cause of ring stains from dried liquid drops. *Nature* **389**, 827-829 (1997).
2. Hu H, Larson RG. Marangoni effect reverses coffee-ring depositions. *J. Phys. Chem. B* **110**, 7090-7094 (2006).
3. Park J, Moon J. Control of colloidal particle deposit patterns within picoliter droplets ejected by ink-jet printing. *Langmuir* **22**, 3506-3513 (2006).
4. Bormashenko E, Stein T, Whyman G, Bormashenko Y, Pogreb R. Wetting properties of the multiscaled nanostructured polymer and metallic superhydrophobic surfaces. *Langmuir* **22**, 9982-9985 (2006).
